# Bidirectional Associations between Objective Physical Activity and Sleep Patterns in Spanish School Children

**DOI:** 10.3390/ijerph17030710

**Published:** 2020-01-22

**Authors:** Manuel Ávila-García, Pedro Femia-Marzo, Francisco Javier Huertas-Delgado, Pablo Tercedor

**Affiliations:** 1 Physical Activity for Health Promotion, Research Group, Faculty of Sport Sciences, University of Granada, Camino de Alfacar 402, 18011 Granada, Spain; tercedor@ugr.es; 2 Unit of Biostatistics, Faculty of Medicine, University of Granada, Avenida de la Investigación 11, 18016 Granada, Spain; pfemia@ugr.es; 3 Physical Activity for Health Promotion, Research Group, La Inmaculada Teacher Training Centre, University of Granada, Calle Joaquín Eguaras 114, 18013 Granada, Spain; fjhuertas@ugr.es

**Keywords:** physical activity, sleep duration, sleep latency, sleep efficiency, accelerometer, children

## Abstract

Physical activity (PA) and sleep contribute to better children’s health. Nonetheless, the bidirectional relationship between both of these health-related factors is unclear when using objective measures. The aims of this study were (1) to describe the PA (light PA and moderate-to-vigorous PA (MVPA) and sleep (duration, latency, and efficiency) patterns of children and compare them with recommendations, and (2) to analyze the bidirectional association between PA levels and sleep patterns in 470 Spanish children according to sex (average age of 8.4 (0.4) years, 51.9% boys). A tri-axial accelerometer and sleep logs were used to measure PA (light PA and MVPA) and sleep patterns (duration, latency, and efficiency) in the children for seven consecutive days. Linear mixed models were conducted to analyze the bidirectional association (PA → sleep and sleep → PA) adjusted for the child, the sex, the school, and the day of observation. The results showed that, overall, the children did not meet the sleep duration recommendations per day. Regarding the bidirectional association, increased light PA and MVPA during the day was related to decreased sleep duration but an improvement in sleep efficiency that night. However, sleep duration and sleep efficiency were only related negatively and positively to light PA the following day, respectively. Regarding sex, light PA was associated with decreased sleep duration in both sexes, although the average value was lower in boys. In addition, light PA was also related only to an improvement in sleep efficiency the same night in both sexes, with girls generally having more efficient sleep. More studies in a representative sample of children that use objective measures to corroborate these results are needed.

## 1. Introduction

The benefits of daily physical activity (PA) and sleep are highlighted by their relationship with improved health in children [1]. For instance, higher PA levels were related to improved cardiorespiratory fitness [2], lower adiposity indicators [3], and better academic performance [4]. Light PA is important to consider because it contributes to improvement in metabolic biomarkers [5], such as the cardiorespiratory system and body composition [6]. Likewise, a systematic review associated optimal sleep quality with lower adiposity indicators, better emotional regulation, academic achievement, and quality of life/well-being [7]. Despite the aforementioned benefits to health, PA has declined globally [8,9], with many not meeting recommendations (60 minutes of moderate-to-vigorous PA (MVPA) per day) [10]. Furthermore, two systematic reviews observed a general decline in sleep duration in children [10,11], resulting in these recommendations (9–11 h per night) not being met either [11]. Nonetheless, sleep latency, defined as the time taken to fall asleep (>30 min per night) [11], and sleep efficiency, defined as the percentage of time in bed spent asleep (≤85% per night) [11], were generally shown to be good in children, with several studies indicating good sleep latency [12,13,14] and sleep quality [15,16,17] per night.

Because both PA and sleep are associated with children’s health, determining whether a bidirectional relationship exists between these two behaviors is generating interest [15]. Over the last decade, various studies analyzed the association using accelerometry [14,15,17,18,19,20,21,22]. However, the bidirectional relationship was not clear due to opposing results; while some studies showed a negative association between both behaviors [18,22], the study carried out by Lin et al. [15] was the only one to show a positive relationship between high PA and long sleep duration the same night. Other studies showed no association [19,22]. High PA was also positively related to better sleep efficiency [21] that night, while Mcneil et al. [17] showed that high sleep efficiency was associated with a decrease in light PA the following day. To our knowledge, the study carried out by Vicent et al. [19] was the only study that did not report any bidirectional association between PA and sleep.

In this study, we considered it important to delve into how sex mediated this association due to previous studies that presented differences in the daily MVPA levels between boys and girls, determining that boys tended to be more physically active [23,24]. We found some studies that analyzed the bidirectional association between PA and sleep in children [15,18,20,21], however, only two studies observed significant differences [18,20]. In the study carried out by Pesonen et al. [18] in Finnish children, sleep duration decreased in both boys (*b* = −0.21) and girls (*b* = −0.38) in terms of MVPA SD units (all *p* < 0.001) the same night. Likewise, another study carried out in Croatian, Slovenian, and American children associated an extra hour spent in bed during the night with 20 minutes less MVPA the following day (*p* < 0.001) in both sexes [20]. We also considered it important to analyze light PA, which is a key factor in health promotion and disease prevention [5,25], due to a few studies that found light PA was related to sleep [15,17,19]. In addition, light PA is the predominant PA level performed in daily life and is easier to modify, thereby contributing to a reduction in sedentary time as well as an increase in PA [25]. Hence, the difference between light PA and MVPA according to sex could explain in more detail the above-mentioned relationship between PA and sleep. Therefore, the aims of this study were (1) to describe the PA (light PA and MVPA) and sleep (duration, latency, and efficiency) patterns of third-grade Spanish children and compare these patterns to recommendations, and (2) to analyze the bidirectional associations between PA (light PA and MVPA) and sleep (duration, latency, and efficiency) according to sex.

## 2. Materials and Methods

### 2.1. Participants

The data were obtained as part of the Previene Project (Promoting Healthy Lifestyles in the School Environment) [26], a quasi-experimental study focused on promoting PA and sleep hygiene in primary schools of Granada (Spain). This project was carried out in various schools for two years. However, the baseline data of this study were collected in two different stages, first, during January–March 2017, and second, during the same period in 2018.

A total of 16 schools were recruited by convenience, inviting a total of 717 third-grade students (8–9 years old) from primary schools in Granada (Spain) to participate in the study. However, 247 students were excluded from data analysis; 142 did not give informed consent, 13 did not attend the evaluation day, 67 did not wear the accelerometer for seven days, and 25 did not deliver the sleep log. The final sample was 470 schoolchildren (average age of 8.4 (0.4) years, 51.9% boys).

The study protocol was approved by the Ethics Committee on Human Research of the University of Granada (Reference: 57/CEIH/2015). After participation approval from the schools, the research team conducted a meeting with the teachers to explain the evaluation process to be performed. The families received an invitation to an initial meeting to receive information and to encourage their participation in the study. Parents signed informed consent forms for the inclusion of their children in the study.

### 2.2. Instruments

#### 2.2.1. Anthropometry

The children’s height and weight measurements were taken to calculate body mass index (BMI), with all children wearing shorts, a short-sleeved shirt, and bare feet for both measurements. Weight was measured with a 0.1 kg approximation using a Seca 876 weighing system (Seca, Ltd., Hamburg, Germany). Height was measured in the Frankfort plane with an approximation of 0.1 cm using a Seca 213 stadiometer (Seca, Ltd., Hamburg, Germany). Height and weight were measured twice, with the averages taken for both measurements. BMI was calculated as weight in kilograms divided by height in meters squared. We used the age and sex BMI cut-off points proposed by the International Obesity Task Force to determine the weight status of each child [27].

#### 2.2.2. Physical Activity and Sleep Data

PA and sleep variables were measured using a tri-axial accelerometer (Actigraph wGT3X-BT, Pensacola, FL, USA), which was considered a valid and reliable tool to objectively measure the PA [28] and quantity/quality of sleep [29] in the children. Participants were instructed to wear an accelerometer attached to their non-dominant wrist for 7 consecutive days, 24 h/day, which offered more accurate data than when the accelerometer was placed at the waist [18]. Parents were also informed to complete a sleep log to determine the time that their children were out of bed, bathing/showering, or involved in other water activities. The data from the sleep log were collated using Actilife software in order to obtain more precise data regarding when the children went to bed and got up. The minimum amount of time considered acceptable for inclusion in the sample was at least 5 days of at least 10 h per day, including 1 weekend day.

The accelerometers were initialized the following day (06:00). Chandler’s algorithm was used to estimate the light PA and MVPA levels [30]. The data were collected at a sampling rate of 90 Hz [31], set to record in 5-s intervals. The cut-off points used to determine the type of intensity were 306–817 counts per 5 s for light PA, 818–1968 counts per 5 s for moderate PA, and >1969 counts per 5 s for vigorous PA.

Furthermore, we evaluated the sleep data using the algorithm proposed by Sadeh et al. [29]. Data were collected set to record in 60-s intervals, with sleep duration (amount of actual time asleep in a sleep episode), sleep latency (amount of time from “lights out,” or bedtime to the onset of sleep), and sleep efficiency (total sleep duration divided by the time lying down in bed and the total sleep time) being the analyzed variables.

### 2.3. Covariates

The sex, the child, the school, and the day of observation were included as covariates in the statistical models.

### 2.4. Data Analysis

Descriptive statistics were reported for all of the measured variables. To examine the differences in PA (light PA and MVPA) and sleep (duration, latency, and efficiency) according to sex, independent *t*-tests were conducted. To analyze the bidirectional association between PA (light PA and MVPA) and sleep (duration, latency, and efficiency), we constructed linear mixed models adjusting all analyses for sex, the child, the school, and the day of observation. The random effects included were the intercepts between the school and the child. The association between PA performed during the day (light PA and MVPA) and sleep outcomes the same night (duration, latency, and efficiency) was analyzed. Then, the association between sleep (duration, latency, and efficiency) and the amount of PA (light PA and MVPA) performed the following day was analyzed. All of the sleep and PA variables were standardized. Hence, the regression coefficients referred to how many standard deviations the response variable changed by per standard deviation increase in the corresponding regressor variable. The level of statistical significance was set at α = 0.05, the confidence level for the intervals was 95%, and all statistical analyses were performed using SPSS (version 23.0; SPSS, Chicago, IL, USA).

## 3. Results

Table 1 shows the descriptive data of the participants. Most participants met the minimum recommended daily MVPA (97%). Boys spent 7.22 min less performing light PA, but performed 6.56 min more MVPA than girls (*p* < 0.05). Regarding sleep patterns, 7% of the students met sleep duration recommendations of 9–11 h per night. It took less than 30 min for 87% of the children to fall asleep, and 38% of the children reached the optimal level of sleep efficiency, i.e., a minimum of 85%. No differences were found in the sleep variables investigated in this study between boys and girls. 

Table 2 shows the association between PA and sleep patterns the same night and sleep patterns and PA the following day. On one hand, light PA and MVPA significantly predicted same-night sleep duration and sleep efficiency. Specifically, sleep duration decreased (*b* = −0.18) and sleep efficiency increased (*b* = 0.19) for each SD unit of light PA (all *p* < 0.05). Meanwhile, sleep duration decreased (*b* = −0.07) and sleep efficiency increased (*b* = 0.07) for each SD unit of MVPA (all *p* < 0.05).

On the other hand, sleep duration and sleep efficiency significantly predicted only light PA the following day. Specifically, light PA decreased (*b* = −0.10) for each SD unit of sleep duration and increased (*b* = 0.06) for each SD unit of sleep efficiency (all *p* < 0.05).

Table 3 shows the association between PA and sleep patterns the same night and sleep patterns and PA the following day according to sex. Light PA significantly predicted sleep duration and sleep efficiency the same night in boys and girls, but sleep latency was significantly predicted only in girls. In boys, sleep duration decreased (*b* = −0.16) and sleep efficiency increased (*b* = 0.16) for each SD unit of light PA (all *p* < 0.001). In girls, sleep duration decreased (*b* = −0.22), while sleep efficiency and sleep latency increased (*b* = 0.22 and *b* = 0.10) for each SD unit of light PA (all *p* < 0.05). MVPA significantly predicted only same-night sleep duration and sleep efficiency in girls. Specifically, sleep duration decreased (*b* = −0.09) and sleep efficiency increased (*b =* 0.08) (all *p* < 0.05).

Sleep duration significantly predicted PA the following day in boys and girls, whereas sleep efficiency was only significantly predicted in boys. Boys exhibited decreased amounts of light PA (*b* = −0.09) for each SD unit of sleep duration and increased amounts of light PA (*b* = 0.09) for each SD unit of sleep efficiency (all *p* < 0.05). Girls exhibited decreased amounts of light PA (*b* = −0.10) for each SD unit of sleep duration (*p* = 0.10).

## 4. Discussion

The purposes of this study were (1) to describe PA (light PA and MVPA) and sleep (duration, latency, and efficiency) patterns in third-grade Spanish children and compare them with recommendations, and (2) to analyze the bidirectional association between PA (light PA and MVPA) and sleep (duration, latency, and efficiency) according to sex.

In our study, the daily MVPA recommendations were met by 97% of the children, which was a higher percentage compared with other international studies in children, where PA was objectively measured with an accelerometer placed on the waist or the hip. In a study in children aged 9–10 from twelve countries, only 44% met the daily minimum MVPA recommendations [32]. Likewise, in another study in children aged 9 and 11 from twelve countries, this percentage was similar, with 44.1% of children meeting MVPA recommendations [33]. However, a study in English and Spanish youths aged 10–14 years showed that only 26.6% of Spanish youths met MVPA recommendations [34]. Conversely, in Australian children of similar ages, Vincent et al. [19] measured PA through accelerometers placed on the wrist, as in our study, showing that 88% of children met the daily MVPA guidelines. These differences in MVPA could be related to the part of the body where the accelerometers were placed [35]. In this sense, a study carried out by Kumahara et al. [36] analyzed PA in children using accelerometers on the wrist and waist. The results showed higher values when the accelerometer was worn on the wrist because of greater acceleration of the upper extremities compared to the movements of the trunk of the body. In our study, boys spent more time performing MVPA than girls, but girls spent more time performing light PA. These results were consistent with the results of another study carried out by Konstabel et al. [24] in children from eight European countries, in which boys were shown to be more physically active than girls (boys 48.5%; girls 9.9%). Specifically, in the case of Spanish children in the same study, boys also spent more time performing MVPA than girls (boys 30.4%; girls 12.3%) [24]. Likewise, in another study in Spanish children, boys spent more time performing MVPA (boys 60%; girls 34.1%) [23]. These differences between the sexes may be influenced by several factors. A recent systematic review observed that the main barriers limiting girls’ participation in sport activities could be related to body image, the biological changes experienced during puberty, lack of motivation caused by perceived low motor skills, fewer PA opportunities, the influence of PE teachers, lower parental support, school work and home responsibilities, and environmental factors [37]. Therefore, it is necessary to develop coeducational interventions to promote PA through games and sports that favor equality between boys and girls with the aim of reducing differences between the sexes.

Regarding the sleep variables, only 7% of the children in our study slept for 9–11 h per day. It took less than 30 min for 87% of the children to fall asleep, and 38% of the children reached the optimal level of sleep efficiency, i.e., a minimum of 85% [11]. In comparison to our results, three studies evaluating children of similar ages from twelve countries showed that 42% [27], 41.9% [33], and 44.1% [15] of children slept for 9–11 h per night, thereby meeting the sleep duration guidelines [38]. However, a study carried out by Lin et al. [15], which was the only one to measure sleep efficiency, showed that 96.2% of children had good sleep efficiency [11]. Vast differences were observed between the percentages obtained in our study with the percentages of the analyzed studies, which may have been caused by the placement of the accelerometer. In our study the accelerometer was placed on the non-dominant wrist, while in the other studies it was placed on the waist or hip; these areas tend to show an overestimation of sleep variables [18]. Shorter sleep duration may have been caused by the increased use of technology before sleeping, such as mobile phones, computers, video games, or television, which affects both sleep duration and quality [39]. A systematic review investigated the determinants of sleep behaviors in longitudinal studies in children aged 4–12 years. Moderate evidence was found regarding the relationship between more screen-time and television before sleeping and short sleep duration and poor sleep quality. Strong evidence was also found regarding a negative association between a child’s age and their sleep duration [40]. In addition, a meta-analysis analyzing the effect of media devices on sleep outcomes observed a strong association between bedtime media device use with short sleep duration and poor sleep quality [41]. According to a systematic review, these relationships could be due to delays before sleeping caused by excessive screen entertainment and increased arousal due to the type of content children tend to view, as well as melatonin suppression induced by screen lights in the evening [42]. Therefore, in order to improve children’s sleep, parents should establish bedtime routines and healthy early sleep habits, including an appropriate and consistent bedtime and reduced screen-time before sleeping [42]. In addition, including activities related to nutrition (e.g., not eating too much during dinner), hygiene (e.g., bathing), and communication (e.g., reading) could also help to improve children’s sleep quality [43].

In our study, both light PA and MVPA were related to shorter sleep duration and improvement in sleep efficiency the same night. Specifically, MVPA reduced sleep duration to a lesser extent than light PA, while light PA improved sleep efficiency more than MVPA. Furthermore, sleep duration and sleep efficiency negatively and positively predicted the amount of time spent performing light PA the following day, respectively. Conversely, Lin et al. [15] showed that both MVPA and light PA were associated with longer sleep duration that night, and light PA increased sleep duration to a greater extent than MVPA. A systematic review investigated how exercise impacted the duration and quality of sleep, concluding that exercise promoted increased sleep duration as well as improved sleep quality in children [44]. It is possible that the children in our study spent more time performing PA in the evening, which may have influenced the sleep duration to be shorter. Pesonen et al. [18] associated periods of more than 30 min of MVPA in the evening with shorter sleep duration in Finnish children aged 7–8 years. Regarding PA and sleep efficiency, Mcneil et al. [17] found that sleep efficiency negatively predicted the amount of light PA performed the following day in Canadian children aged 9–11 years old. However, in our study, PA improved sleep efficiency. The regular practice of PA could help to reduce nocturnal movements and awakenings, thus improving sleep efficiency [17]. A study of Swiss adolescents associated an increase in PA with a low number of awakenings, thereby improving their sleep efficiency [45]. Additionally, the hormone cortisol is related to PA [46] and sleep efficiency [17,47]. Specifically, a study by Martikainen et al. [46] showed that physically active children had lower levels of cortisol. According to this, higher levels of PA may have helped the children in our study reduce their cortisol levels and improve their sleep efficiency.

In relation to sex, light PA was associated with shorter sleep duration in boys and girls, however, this reduction was less in boys. Light PA was also related to in sleep efficiency the same night in both sexes, with girls exhibiting better efficiency. MVPA it was only related to shorter sleep duration and sleep efficiency improvement the same night in girls. Likewise, in a study carried out by Pesonen et al. [18], PA was related to shorter sleep duration in both sexes, although this value was lower in boys. Soric et al. [20] found that spending 60 min longer in bed was related to performing 16 min less MVPA the following day in children from Croatia, Slovenia, and the USA (10–12 years old), but only in girls. Consequently, more time spent performing light PA could play a more important role in improving sleep efficiency in girls [15]. Nonetheless, more studies are needed to corroborate these results and to establish precise conclusions due to the differences between the sexes.

This study offers new outcomes regarding the bidirectional relationship between PA and sleep in children using accelerometry and a sleep log in order to better understand the relationship between both variables according to the differences between the sexes. To our knowledge, this is the first study in Spanish children where sleep was evaluated using an accelerometer placed on the wrist, thereby allowing more accurate measurement of sleep, although also indicating higher PA levels. Another limitation of this study was that schools were recruited by convenience. Hence, we cannot generalize our results to the general population.

## 5. Conclusions

Our results showed that, although most of the children met the daily PA guidelines, most did not meet sleep recommendations. In addition, PA was shown to be bidirectionally related to shorter sleep duration and improvement in sleep efficiency. Concerning sex differences, boys were more physically active than girls, however, light PA contributed to a greater improvement in girls’ sleep patterns. This study provided results obtained through objective measurements in order to better understand the relationship between PA and sleep, as well as to investigate the differences according to sex laid out in the scarce studies found in the literature. Thus, this study could also contribute to the development of school intervention programs aimed to improve children’s PA and/or sleep patterns while considering the differences between boys and girls. Nonetheless, more national and international studies in children using objective measures are needed to corroborate these findings.

## Figures and Tables

**Table 1:** Descriptive data of participants.

	Total Mean (SD)	Boys’ Mean (SD)	Girls’ Mean (SD)	*p*-Value
n	470	244	226	
Age (years)	8.35 (0.32)	8.36 (0.32)	8.34 (0.32)	0.649
Weight (kg)	29.73 (5.27)	29.99 (5.22)	29.45 (5.33)	0.270
Height (m)	1.32 (0.06)	1.33 (0.06)	1.31 (0.06)	**0.026**
BMI (kg)	16.97 (2.10)	16.97 (1.98)	16.98 (2.24)	0.956
**PA**				
Light PA (min/day)	235.13 (30.30)	231.66 (30.59)	238.88 (29.60)	**0.010**
MVPA (min/day)	107.53 (28.04)	110.68 (28.29)	104.12 (27.43)	**0.011**
**SLEEP**				
Sleep duration (min/day)	489.43 (37.09)	489.30 (39.50)	489.58 (34.39)	0.935
Sleep latency (min/day)	17.10 (11.56)	17.13 (10.63)	17.07 (12.50)	0.956
Sleep efficiency (%)	82.74 (5.79)	82.75 (5.86)	82.73 (5.73)	0.972

SD: standard deviation; BMI: body mass index; PA: physical activity; MVPA: moderate-to-vigorous physical activity; Significant values are highlighted in bold.

**Table 2:** Association between (**a**) PA and sleep patterns the same night and (**b**) sleep patterns and PA the following day.

		*B* (95% CI)	*p*-Value
(a) PA → SLEEP			
Light PA (min)	Sleep duration (min)	−0.18 (−0.24, −0.13)	**<0.001**
	Sleep latency (min)	0.04 (−0.00, 0.09)	0.060
	Sleep efficiency (min)	0.19 (0.13, 0.25)	**<0.001**
MVPA (min)	Sleep duration (min)	−0.07 (−0.12, −0.01)	**0.017**
	Sleep latency (min)	0.01 (−0.03, 0.06)	0.534
	Sleep efficiency (min)	0.07 (0.02, 0.13)	**0.013**
**(b) SLEEP → PA**			
Sleep duration (min)	Light PA (min)	−0.10 (−0.15, −0.05)	**<0.001**
	MVPA (min)	0.03 (−0.02, 0.09)	0.245
Sleep latency (min)	Light PA (min)	0.01 (−0.04, 0.07)	0.636
	MVPA (min)	−0.01 (−0.06, 0.06)	0.968
Sleep efficiency (min)	Light PA (min)	0.06 (0.00, 0.11)	**0.025**
	MVPA (min)	−0.01 (−0.06, 0.05)	0.833

*b* = regression coefficients; CI: confidence interval; PA: physical activity; MVPA: moderate-to-vigorous physical activity; Significant values are highlighted in bold.

**Table 3:** Association according to sex between (**a**) PA and sleep patterns the same night and (**b**) sleep patterns and PA the following day.

		BOYS		GIRLS	
		*b* (95% CI)	*p*-Value	*b* (95% CI)	*p*-Value
(a) PA → SLEEP					
Light PA (min)	Sleep duration (min)	−0.16 (−0.23, −0.09)	**<0.001**	−0.22 (−0.31, −0.13)	**<0.001**
	Sleep latency (min)	0.01 (−0.05, 0.06)	0.902	0.10 (0.03, 0.18)	**0.008**
	Sleep efficiency (min)	0.16 (0.09, 0.23)	**<0.001**	0.22 (0.13, 0.32)	**<0.001**
MVPA (min)	Sleep duration (min)	−0.05 (−0.12, 0.03)	0.211	−0.09 (−0.17, −0.02)	**0.015**
	Sleep latency (min)	0.00 (−0.06, 0.06)	0.978	0.04 (−0.03, 0.10)	0.240
	Sleep efficiency (min)	0.06 (−0.01, 0.14)	0.105	0.08 (0.00, 0.16)	**0.049**
**(b) SLEEP → PA**					
Sleep duration (min)	Light PA (min)	−0.09 (−0.16, −0.02)	**0.016**	−0.10 (−0.17, −0.02)	**0.010**
	MVPA (min)	0.04 (−0.03, 0.11)	0.273	0,01 (−0.08, 0.10)	0.795
Sleep latency (min)	Light PA (min)	−0.02 (−0.10, 0.07)	0.723	0.04 (−0.03, 0.12)	0.240
	MVPA (min)	0.01 (−0.07, 0.09)	0.866	−0.01 (−0.09, 0.07)	0.781
Sleep efficiency (min)	Light PA (min)	0.09 (0.01, 0.16)	**0.018**	0,03 (−0.04, 0.10)	0.352
	MVPA (min)	−0.01 (−0.08, 0.06)	0.804	−0,01 (−0.09, 0.07)	0.804

*b*: regression coefficients; CI: confidence interval; PA: physical activity; MVPA: moderate-to-vigorous physical activity; Significant values are highlighted in bold.
